# The advances of DNA methylation in the liquid biopsy for the detection of lung cancer

**DOI:** 10.3389/fonc.2025.1547797

**Published:** 2025-07-01

**Authors:** Yuxuan Chen, Shuang Peng, Cailian Wang

**Affiliations:** ^1^ School of Medicine, Southeast University, Nanjing, Jiangsu, China; ^2^ Department of Oncology, Zhongda Hospital, School of Medicine, Southeast University, Nanjing, Jiangsu, China

**Keywords:** liquid biopsy, DNA Methylation, lung cancer, biomarker, CtDNA

## Abstract

Lung cancer is the most common cancer in the world and the leading cause of cancer death. The absence of effective early detection is one of the major contributors to high mortality rate of lung cancer. Liquid biopsy has the potential to become as a new method for early detection of cancer due to its non-invasive nature, ease of access, and overall presentation of tumor. Liquid biopsy has garnered increasing attention for its role in early detection and tumor genome assessment through the examination of circulating tumor DNA (ctDNA) released by apoptotic or necrotic tumor cells. DNA methylation is a potential biomarker for liquid biopsy due to its early onset, cancer specificity, biological stability, and accessibility in bodily fluids. This review aims to present an overview of the process of DNA methylation, identify potential methylation gene targets, and explore the application of liquid biopsy in the detection of lung cancer.

## 1 Introduction

Lung cancer is the most common cancer in the world, it is the leading cause of cancer death (1). The high mortality of the patients with lung cancer is attributed to the fact that over 75% of the patients are diagnosed at an advanced stage (2). The low-dose computed tomography (LDCT) remains the preferred method for early detection of lung cancer due to its high sensitivity of 93.7% (3, 4). However, the high sensitivity comes with a significant false-positive rate of 96.4%, which necessitates a long-term follow-up CT review or even invasive biopsy procedure for confirmation (2). Tissue biopsies to definite early-stage tumors may be difficult because of the anatomical location of the tumor. The discovery of a more accurate diagnostic biomarker is essential to mitigate the unwarranted financial and psychological burden on the patients.

Epigenetic alterations exhibit great stability in cancers, making it a promising candidate for biomarker development (5). DNA methylation plays an important role in the development of tumors, which modulates genetic expression without altering the DNA sequence (6). The advantages of DNA methylation are early onset, cancer specificity, biological stability and accessibility in bodily fluids, making it a more suitable marker compared to the gene variation (7–9). Liquid biopsy based on DNA methylation is a non-invasive test, which only needs a small volume of biological fluid, such as peripheral blood, urine and sputum (10). The aberrant DNA methylation in the early stages of lung cancer can be detected in circulating tumor DNA (ctDNA)and could be a valuable biomarker for the early diagnosis of lung cancer (11). Circulating tumor-derived DNA bear methylation states which can resemble the tumor tissue and can enable the screening and localization of cancers (12).

## 2 DNA methylation and cancer

**Figure 1:**
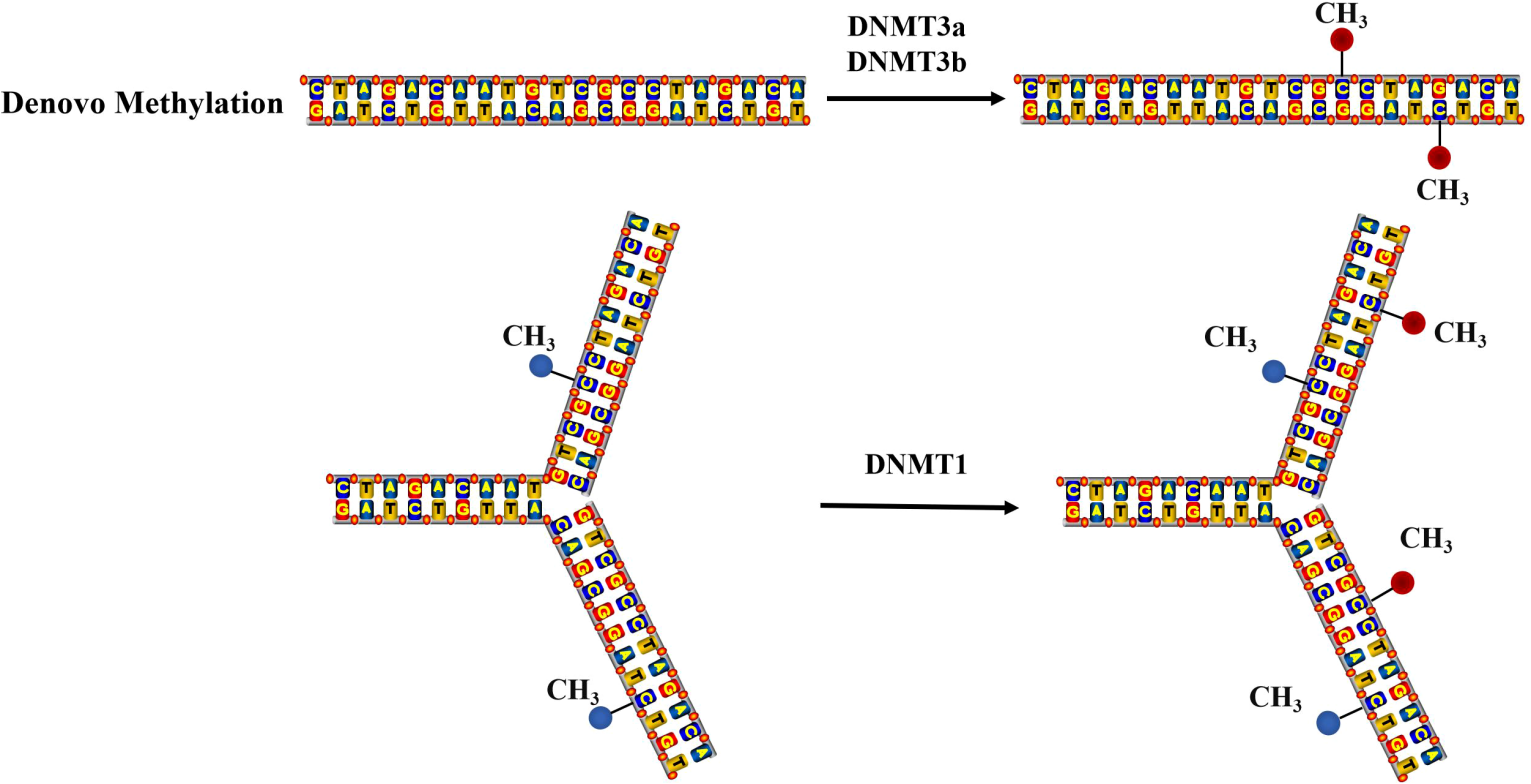
The mechanism of DNA methylation. Denovo methylation is the process of transferring methyl groups to DNA that has not been methylated. Maintenance methylation can methylate an unmethylated strand of double-stranded DNA, when another strand is already methylated.

DNA methylation is facilitated by the enzyme DNA methyltransferase (DNMT), which can selectively transfer methyl groups from S-adenyl methionine (SAM) to the cytosine residues within a DNA sequence, this results of which in the formation of 5-methylcytosine(5-mC) with a minor presence of N6-methylpurine (N6-mA) and 7-methylguanine (7-mG). Denovo methylation and maintenance methylation are two types of DNA methylation mediated by different methyltransferases. Maintenance methylation mediated by DNMT1 involves methylating an unmethylated strand of double-stranded DNA while the other strand is already methylated, which can maintain DNA methylation during replication (13). DNMT1 can bind the replication site and precisely replicates the original DNA methylation pattern by adding methyl groups to the newly synthesized daughter strand during semi-conservative DNA replication, shown as **Figure 1** (14). Denovo methylation mediated by DNMT3a and DNMT3b refers to the transfer of methyl groups onto DNA sequences that have not previously undergone methylation (15).

**Figure 2:**
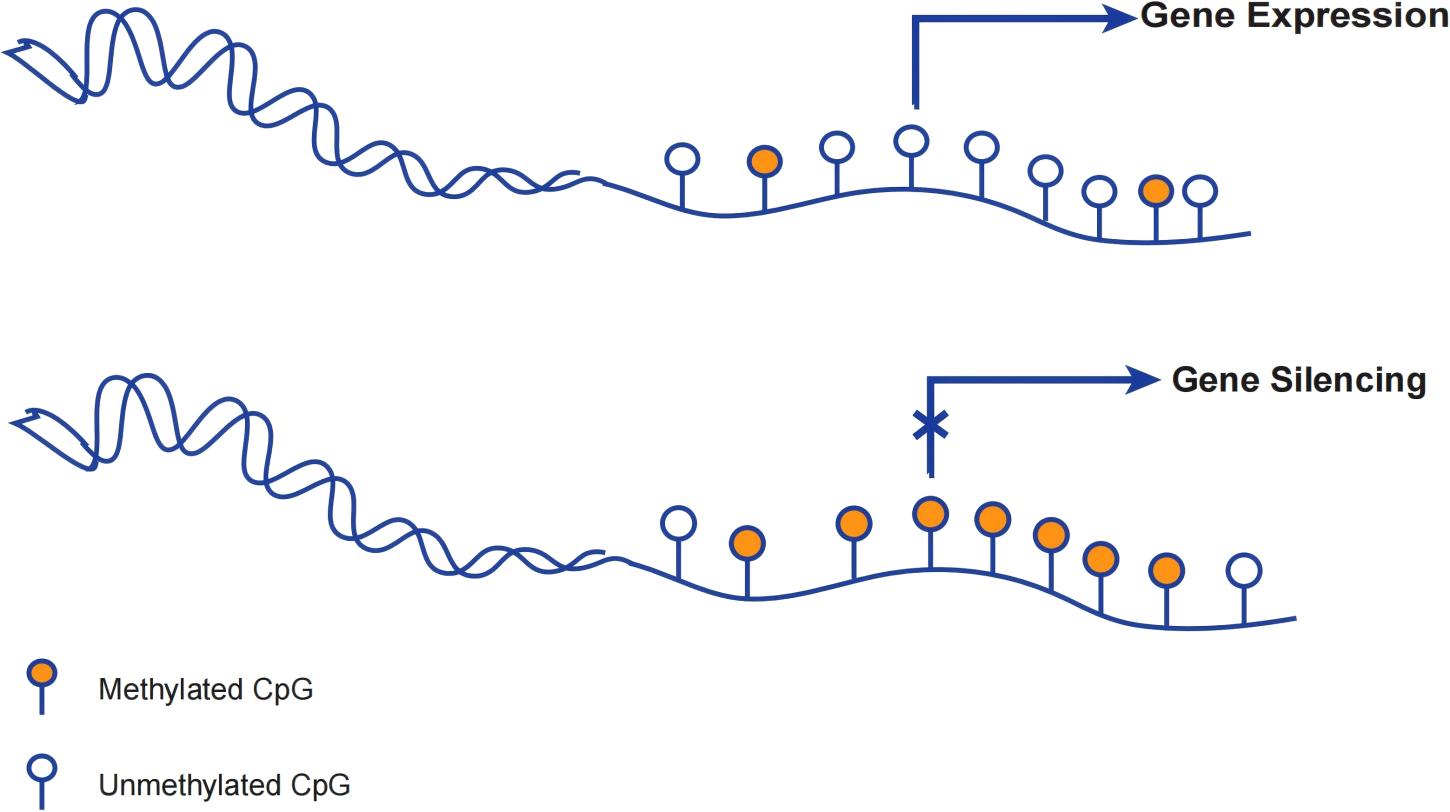
DNA methylation regulating gene expression. When the promoter is unmethylated, the gene can still undergo transcription despite methylation of the downstream bases. Gene silencing occurs when the promoter and transcription start site of a gene are methylated and cannot be properly transcribed at the start site.

DNA methylation generally occurs at the Cytosine phosphate guanine (CpG) dinucleotide site. DNA methylation occurring at CpG sites within promoters will disrupted the transcription which leads to gene silencing (16). (**Figure 2**) Aberrant DNA methylation can lead to dysregulation of gene expression, ultimately resulting in genetic disorders or even cancer (17).Furthermore, the methylation of non-promoter sites also can regulate various essential processes, such as splicing, transcript variations arising from alternative promoters, and activated enhancers (18).

DNA methylation plays an important role in the pathogenesis and progression of lung cancer. Denovo methylation induces cellular mutations and initiates a series of programmed changes in gene expression. In normal cells, most CpG sequences in the genome are methylated, but CpG islands and the nearby CpG island shores (the region within 2 kb of the islands) exhibit a distinct state of hypomethylation (19, 20). The findings of various studies have demonstrated that tumor cells exhibit aberrant DNA methylation patterns: regions with low CpG density display reduced expression of DNA methylation, whereas CpG islands are hypermethylated (20, 21). A hypothesis suggests that once this pattern is formed, it is stably maintained in the descendant cells (22). Meanwhile, this characteristic becomes more pronounced as cancer progresses (23, 24). The entry of DNMT into the nucleus results in the methylation of previously hypomethylated CpG islands, leading to the silencing of tumor suppressor genes, while hypomethylation contributes to the activation of oncogenes. This epigenetic modification leads to diminished cell differentiation, heightened cell proliferation, aberrant apoptosis, angiogenesis, impaired cell adhesion and other cellular dysfunctions that culminate in tumorigenesis (16).

## 3 DNA methylation as a potential biomarker for lung cancer

Aberrant DNA methylation is closely associated with cellular dysfunctions in lung cancer, such as DNA repair (O6 methylguanine DNA methyltransferase, *MGMT*), cell growth (Short State Homebox2, *SHOX2*), and cell cycle (cycling dependent kinase inhibitor 2A, *CDKN2A*). Therefore, it is expected to become a biomarker for evaluating disease status and therapeutic efficacy. Here we will introduce several widely studied and potentially available gene locus.

### 3.1 Death-associated protein kinase

The Death-associated protein kinase (*DAPK*), functioning as a potential tumor suppressor gene, exhibits the capacity to induce apoptosis and impede tumorigenesis (25). *DAPK* inhibits the growth of cancer by promoting cell apoptosis and autophagy. Previous studies have reported that the expression of promoter methylation of DAPK in liquid biopsy is more frequently observed in lung cancer patients than in normal controls (26, 27). The evidence suggests that *DAPK* methylation is an independent prognostic factor, unaffected by age, gender, smoking status, clinical stage, pathological type, or tumor differentiation status (28). This indicates its potential as a biomarker for diagnosing lung cancer.

### 3.2 Multiple tumor suppressor 1

Multiple tumor suppressor 1*(MTS1)*, also known as *P16*, is a tumor suppressor gene involved in cell cycle regulation (29). The function of *P16* is to inhibit the cell cycle by combining with cell Cyclin-dependent kinase (CDKs). Compared with normal lung tissue, NSCLC patients exhibit a higher incidence of *P16* promoter methylation in tumor tissue (30). Furthermore, promoter methylation of *P16* is also an independent factor in NSCLC patients, regardless of tumor stage, age, sex, race, smoking history, and histological characteristics (30).In addition, it is discovered that *P16* promoter methylation is more highly expressed in the plasma of lung cancer patients than in healthy controls (31). This provides a potential non-invasive way to detect lung cancer (32).

### 3.3 Ras association domain-containing protein 1A

Ras association domain-containing protein 1A *(RASSF1A)* is expressed in normal tissues, targeting microtubules and participating in growth regulation. RASSF1A has been proposed as a tumor suppressor, which binds to RAS in the form of GTP and promotes apoptosis (33). In addition, *RASSF1A* is prone to inactivation in the Hippo pathway, leading to excessive tissue growth and cancer occurrence (24, 34). The methylation of *RASSF1A* leads to gene silencing, promotes the process of epithelial mesenchymal transition, and facilitates the acquisition of stemness. It is reported that the level of *RASSF1A* promoter hypermethylation in sputum and bronchial fluid is significantly higher among smokers compared to non-smokers, contributing to an increased incidence of lung cancer (35, 36). Researchers developed a diagnostic model for lung cancer based on methylation status, including *RASSF1A* promoter hypermethylation in plasma, which achieved a high sensitivity (73%) and specificity (82%) (37). These suggest that regular test of *RASSF1A* promoter hypermethylation in sputum, bronchial fluid, or plasma from high-risk populations can aid in early diagnosis of lung cancer.

### 3.4 O (6)-Alkylguanine-DNA alkyltransferase

O6 methylguanine-DNA methyltransferase (*MGMT*) is a DNA repair gene. It protects cells from proteolysis by removing alkyl groups from the O6 of Guanine nucleotides. The MGMT protein is considered as the primary cellular defense mechanism, safeguarding the body against toxicity, mutagenicity, and carcinogenesis resulting from DNA alkylation at the O6 position (38). The animal models have demonstrated that the absence of *MGMT* activity renders a higher susceptibility to alkylation-induced cancer, whereas its overexpression confers a protective effect (39, 40). A meta-analysis combining 20 studies (including 1539 NSCLC patient tissues and 1052 normal or adjacent tissue samples) proposes that: 1) the *MGMT* promoter methylation level in NSCLC tissues is much higher than that in normal tissue samples, and *MGMT* methylation is not related to clinical pathological characteristics such as age, gender, smoking and pathological type; 2) *MGMT* promoter methylation level in tissues from NSCLC patients was higher in late stage (III and IV) than in early stage (I and II) (41). Another research also reported that the rate of *MGMT* promoter methylation in the plasma and bronchoalveolar lavage fluid (BLAF) of lung cancer patients is significantly higher than that of healthy individuals. It also has a high specificity, which can be contributed to accurately diagnosis for lung cancer (42).

### 3.5 Short stature homobox 2

Short State Homebox 2 (*SHOX2*) is a widely transcriptional factor, which is closely related to organ development. It promotes tumorigenesis, epithelial-mesenchymal transition (EMT), bone metastasis, and drug resistance in lung cancer by modulating the expression of downstream target genes. As is reported in previous study, overexpression of bone Morphogenesis protein 4 (Bmp4) and indirect inhibition on RUNX2 expression level can be observed in SHOX2 deficient mice (43). Runx2 has been identified as a mesenchymal stem marker for lung cancer. Meanwhile, SHOX2 is also a novel EMT inducer. Ectopic *SHOX2* expression can reverse EMT related protein level (including catenin, N-cadherin, E-cadherin, and Vimentin), inducing cancer proliferation and metastasis (44). A study containing 172 patients showed a significant difference of *SCOX2* promoter methylation between lung cancer patients and normal controls. With 90% fixation specificity, the sensitivity of LC was 67%. When the fixed sensitivity is 90%, the specificity is 73% (45). The effective early-stage diagnosis of single SHOX2 promoter methylation remains challenging when compared to advanced lung cancer (46). Fortunately, advanced products integrating SHOX2 with RASSF1A have been developed for the detection of dual gene methylation. The sensitivity of detecting alveolar lavage fluid has reached 71.5-83.2%, while the specificity has increased to 90.0-97.4% (47, 48). Therefore, comparing with traditional cytology, the combined detection of SHOX2 and RASSF1A methylation improved the diagnostic efficacy of lung cancer.

### 3.6 Prostaglandin E receptor 4

Prostaglandin E receptor 4 (*PTGER4*), a member of the G protein-coupled receptor family, functions as a significant tumor suppressor (49). Prostaglandin E2 (PGE2) is the most abundant prostaglandin found in lung cancer, and PTGER4 is one of its receptors (50, 51). A validation study demonstrated that the combined SHOX2 and PTGER4 methylation assay is capable of effectively distinguishing lung cancer patients from healthy individuals. At a fixed specificity of 90%, sensitivity for LC was 67%; at a fixed sensitivity of 90%, specificity was 73% (52). The integration of multiple biomarkers with high stage-specificity and histological type specificity, including SHOX2 and PTGER4 DNA methylation as well as IDH1, demonstrated superior diagnostic performance in the detection of lung cancers compared to single-marker assessments (sensitivity=86.1% and specificity = 80.0%) (53). Greatly, a diagnostic kit that targets SHOX2 and PTGER4 using MethyLight technology has received FDA approval, offering a novel method for the early detection of lung cancer (54).

## 4 Liquid biopsy based on DNA methylation

Liquid biopsy is a real-time detection of tumor cells or tumor cell products such as circulating nucleic acids (circulating tumor DNA, ctDNA), circulating tumor cells (CTCs) and exosomes that are released from primary or metastatic tumor lesions into blood or other body fluids (including urine, ascites, pleural effusion, etc.). With the rapid advances of molecular biology and its expending clinical applications, liquid biopsy has emerged as a potential method to compensate for the frequent false-positive results of LDCT (55). Liquid biopsy, as an emerging diagnostic method in recent years, has attracted widespread attention due to its non-invasive nature and higher sensitivity and specificity compared to traditional tumor markers (55, 56).

Liquid biopsies based on DNA methylation can accurately identify the organ of origin of ctDNA and can classify its subtypes. Liquid biopsy based on DNA methylation targets ctDNA, which contains valuable information regarding the tissue of origin. In healthy individuals, most cell-free DNA molecules in plasma originate from blood cells (57, 58). In some pathological conditions, an elevation in cell death within a specific organ or tissue leads to a corresponding increase in the quantity of cfDNA derived from the affected organ or tissue (12). DNA methylation profiles differ between cells of different tissues of origin. Thus, while detecting the presence of cancer, CpG methylation patterns in ctDNA also provide information about the organ site of cancer (59, 60). It was reported that methylation testing achieved a 96% accuracy rate in identifying organ sites (60). Differences in DNA methylation can also distinguish lung cancer subtypes (61, 62). For the identification of lung cancer subtypes, conventional approaches primarily include mRNA expression signatures and multiplex immunohistochemistry (IHC). However, there are some limitations such as mRNA degradation during specimen preservation and subjectivity of IHC detection. Guidelines published by European Society for Medical Oncology (ESMO) in 2022 recommend the test of ctDNA as an alternative to tissue genotyping (63). Existing studies has demonstrated that multiple gene promoter methylation occurs at different methylation frequencies in SCLC and NSCLC. For example, the frequency of *APC* and *P16* methylation in NSCLC was significantly higher compared to that in SCLC (64, 65). While the frequency of *CASP8* and *TNFRSF6* methylation exhibited an opposite trend (66). For NSCLC, through the analysis of public databases, researchers identified that LUAD and LUSC exhibited 391 genes with opposing methylation patterns compared to normal tissues (67). For SCLC, a DNA methylation classifier called SCLC-DMC was proposed with an accuracy of 95.8%(95% CI: 78.9% - 99.9%; Kappa = 0.9286) (61). Thus, DNA methylation holds significant promise for the early detection of lung cancer. The ctDNA methylation profile in the blood of lung cancer patients exhibits high specificity. Biomarkers of DNA methylation such as *SHOX2*, *RASSF1A* and *PTGER4* can be detected during the early stages of lung cancer, achieving both high sensitivity and specificity. Compared with conventional imaging techniques and traditional tumor markers, liquid biopsy based on methylation analysis is capable of detecting tumor indicators at an earlier stage and may even predict risks prior to the appearance of evident lesions on imaging (68).

Thus, DNA methylation has great potential in the early detection of lung cancer. The ctDNA methylation profile in the blood of lung cancer patients is highly specific. Some methylation markers, such as SHOX2, RASSF1A, APC, etc., can be detected in the early stage of lung cancer, with both sensitivity and specificity reaching a relatively high level. Compared with traditional imaging examinations and tumor markers, liquid biopsy based on methylation can detect tumor signs earlier and even indicate risks when obvious lesions have not yet appeared on imaging.

Sputum and blood are samples for liquid biopsy of lung cancer, both of which are readily available. Studies have shown that sputum, as a liquid biopsy sample for DNA methylation, exhibits greater sensitivity but lower specificity compared to blood (69, 70). The presence of microbial DNA and other non-target molecules in the sputum can affect the accuracy of results. Some specific techniques and methods to optimize the processing and extraction of sputum samples may be necessary to improve the specificity of detection. Smoking causes changes in the genetic material of bronchial squamous epithelial cells, forming the extensive field cancerization, which leads to false-positive results when sputum is used as a sample (71). The advantage of blood as a sample is that false positives caused by this phenomenon can be avoided (28, 30). However, if DNA released by tumors is too fragmented to detect the methylation, it results in lower sensitivity when plasma is used as a sample (35). Therefore, sputum and blood as samples for liquid biopsies can be applied at the same time to achieve higher detection rates, if it is economically feasible.

The advantages of liquid biopsy based on DNA methylation include early onset, cancer specificity, and biological stability. Firstly, DNA methylation takes place at an early stage of the tumor. Published studies with large sample sizes have demonstrated the potential of liquid biopsy based on DNA methylation in effectively detecting cancers at early stages, such as those in stage I or asymptomatic stages, while maintaining a minimal false positive rate (60, 72). Secondly, unlike mutations as markers that can occurs in the normal tissues (73), DNA methylation patterns between normal and cancer cells are widespread differences. The level of DNA methylation in tumor cells is reduced in regions with low CpG density, and the CpG islands are hypermethylated, which is different from normal cells. Meanwhile, methylation patterns of closely situated CpG sites tend to exhibit similarity, referred to as methylation haplotype blocks (59). Methylation haplotype blocks, when displaying distinct methylation states in tumor and non-tumor DNA, are easier to distinguish compared to other tumor DNA features, such as point mutations. Thirdly, DNA methylation is a stable alteration that can be efficiently and accurately quantified through methylation-specific PCR(MSP) techniques (11).

The accuracy of liquid biopsy based on a single methylation site may be limited. (**Table 1**) Therefore, a formula combining multiple methylation sites has been developed (74–76). For example, a study suggested that the corresponding methylation index (IM) was calculated based on the methylation status of several genes in ctDNA bound to plasma and cell membrane (77). It was reported that the IM values of *RASSF100A* and *RARB1* in lung cancer patients increase 2-3 times compared with healthy individuals, which may help early detection of lung cancer. The comprehensive evaluation of multiple DNA methylation targets can greatly enhance the sensitivity and specificity of detection.

**Table 1:** Sensitivity and specificity of DNA methylation for lung cancer detection.

Gene	Sample	Sample size	Detection	Sensitivity	Specificity	Reference
DAPK	Sputum	72	Nested methylation-specific PCR	45%	76%	(69)
	Plasma	72		9%	90%	(69)
DAPK, CT	Plasma	92	Methylation-specific PCR	87%	82%	(78)
P16	Sputum	72	Nested methylation-specific PCR	62%	79%	(69)
	Plasma	72		18%	76%	(69)
DAPK, P16, Runx3	Plasma	60	Methylation-specific PCR	92%	85%	(79)
MGMT	Sputum	72	Nested methylation-specific PCR	45%	70%	(69)
	Plasma	72		27%	98%	(69)
MGMT, CT	Plasma	92	Methylation-specific PCR	93%	92%	(78)
RASSF1A	Sputum	72	Nested methylation-specific PCR	29%	76%	(69)
	Plasma	72		16%	95%	(69)
	Plasma	305	Methylation-specific PCR	50.4%	96.2%	(47, 48)
SHOX2	Sputum	69	Methylation-specific PCR	83%	84%	(70)
	Plasma	305	Methylation-specific PCR	64.2%	92.3%	(47, 48)
RASSF1A, SHOX2	Plasma	305	Methylation-specific PCR	71%	90%	(47, 48)
	Plasma	322	Methylation-specific PCR and sanger sequencing	81%	97%	(47, 48)
SHOX2, PTGER4, IDH1	Plasma	221	Methylation-specific PCR	86%	80%	(53)

## 5 Promises and challenges

Liquid biopsy based on DNA methylation shows great potential as a biomarker for the early detection of lung cancer. On the one side, liquid biopsy based on DNA methylation is non-invasive, inexpensive and repeatable, offering significant advantages in assisting the early detection of lung cancer and providing a reliable risk assessment for the management of uncertain pulmonary nodules (IPNs) in high-risk populations (19). For example, a model proposed recently called LUNG-TRAC assesses the risk of IPNs by detecting abnormal methylation of ctDNA in the blood, which achieved an area under the curve (AUC) of 0.810 (sensitivity=74.4% and specificity= 73.7%) (80). Another model, named PulmoSeek, was designed to differentiate between benign and malignant lung nodules, achieving an accuracy rate of 80.0% among 140 samples (81). Researchers have suggested that combining ctDNA methylation biomarkers with conventional lung cancer risk factors can improve the accuracy of identification (82). The PulmoSeek Plus model, which combines the PulmoSeek model and clinical characteristics, classifies lung nodules with two cutoffs (0.65 and 0.89) and can reduce unnecessary surgery by 89% (105/118) and delayed treatment by 73% (308/423) (68). Compared with the previously proposed models based on patient clinical and radiological characteristics, such as the Mayo Clinic model (“ Mayo “) (83) and the Veterans Administration (“ VA “) model (84), these models showed better sensitivity and specificity. On the other side, ctDNA contains information related to its tissue of origin, offering valuable biological information about the primary tumor (11). The detection of ctDNA methylation can improve the limitations of tissue biopsy that may may fail to fully reflect tumor heterogeneity (11, 85).

Although the application of liquid biopsies based on DNA methylation has great potential in the detection of cancer, there are still some limitations. A major challenge in ctDNA detection comes from DNA interference from normal blood cells, like clonal hematopoiesis. 53.2% of ctDNA mutations resulted from an increase in clonal hematopoiesis (86). To reduce the false positive results of ctDNA detection caused by clonal hematopoiesis, it is recommended to perform concurrent analysis of plasma ctDNA and leukocyte DNA to exclude mutations arising from clonal hematopoiesis (63, 86). On the other side, false negatives also represent a significant challenge in the detection of ctDNA methylation. Potential causes include the low concentration of ctDNA in plasma, inadequate sensitivity of the detection methods, or the possibility that the tumor does not release detectable levels of ctDNA (63). Firstly, ctDNA in the plasma of cancer patients is predominantly composed of short fragments, less than 200 base pairs (87, 88), and the half-life of ctDNA in cancer patients appears to be less than two hours (89, 90). These factors may contribute to the low concentration of ctDNA in plasma. Employing shorter PCR amplicons, such as those under 100 base pairs, enables the measurement of a higher relative concentration of ctDNA (87, 91). In instances where timely ctDNA testing is not feasible, samples should be stored at -80°C and the frequency of freeze-thaw cycles should be minimized. Secondly, current ctDNA methylation analysis for diagnostics predominantly utilize two methodologies: PCR-based approaches or next-generation sequencing (NGS). The sensitivity and specificity will be greatly reduced, if the cfDNA fragment is too small (87, 88). The detection of DNA methylation requires high sensitivity to variant Allele frequency (VAF) detection between 0.1% and 0.01% in order to reliably predict the probability of detection (92). Meanwhile, the cost of repeating the examination to detect each biomarker is higher, while multiplex assays also presents challenges related to PCR mixtures such as primer-dimers and PCR competition (93). It poses a higher challenge for the detection. As technology advances, these limitations may be improved. For example, droplet digital PCR (ddPCR) can detect VAF ≤ 0.01%, and has the advantages of low cost, fast speed and high sensitivity (94). Multiplex digital methylation-specific PCR (mdMSP) which was proposed in recent years, not only retains the advantages of digital droplet PCR (ddPCR) but also demonstrates superior sensitivity and specificity(sensitivity = 90% and specificity =82%) (93). Thirdly, due to the inevitable false negative potential of ctDNA testing, reflex tumor testing should be considered when the test results of ctDNA are negative (63).

## 6 Conclusion

Methylated biomarkers are proved to be effective for early diagnosis of lung cancer. It is believed that liquid biopsy based on DNA methylation will make outstanding contributions to the early diagnosis of LC in the future.
